# The Bogus Diploma Business in Pennsylvania

**Published:** 1881-02

**Authors:** 


### THE BOGUS DIPLOMA BUSINESS IN PENNSYLVANIA.

The Governor of this State, in his annual message, given to the public last week, presents a succinct resume of what was done by the authorities to check the disgraceful traffic in diplomas in this Commonwealth. He states that the General Assembly requested the Attorney General to institute proceedings in the Courts against the American University of Philadelphia, on account of the abuse of its franchises "in the unlawful sale of diplomas to persons who had not pursued the prescribed course of study, and who were unfitted, by reason of ignorance, to practice medicine." In accordance with this request writs of quo warranto were sued out by him against the American University of Philadelphia and the Eclectic Medical College of Pennsylvania, another institution claiming a like charter, and managed by the same persons controlling the American University. In both cases favorable judgments have been rendered, and the said charters declared forfeited by the Courts, and as an incidental result, the manager and official head of these institutions has been convicted and imprisoned for violating the laws relative to the sale of diplomas and other crimes. Proceedings, not yet terminated, have also been commenced against the Philadelphia University of Medicine and Surgery.

This action on the part of the State authorities has not been too speedy. Evidence of the sale of diplomas in large numbers, not only in this country but in England and on the Continent of Europe, has been secured, and complaints transmitted through our representatives abroad to the State Department at Washington, of the presence in the hands of grossly ignorant persons of regularly issued diplomas from institutions chartered by the State of Pennsylvania, authorizing the holders to practice medicine, are corroborative of ascertained facts. It was high time that so serious a disgrace to the good name of the State should be abated.

No appropriation was made to enable the Attorney General to prosecute this work, and he was practically powerless until the necessary means were advanced by a public-spirited citizen of Philadelphia, to whom, it is hoped, recompense will be speedily made, through appropriate legislation. The Governor recommends the adoption of a system of registration of medical diplomas, with appropriate penalties for non registration or for practicing under a diploma irregularly obtained.—*Med and Surg. Reporter.*

